# Synergistic Effect of Mesoporous Silica and Hydroxyapatite in Loaded Poly(DL-lactic-co-glycolic acid) Microspheres on the Regeneration of Bone Defects

**DOI:** 10.1155/2016/9824827

**Published:** 2016-08-29

**Authors:** Shu He, Kai-Feng Lin, Jun-Jun Fan, Gang Hu, Xin Dong, Yi-Nan Zhao, Yue Song, Zhong-Shang Guo, Long Bi, Jian Liu

**Affiliations:** ^1^Department of Orthopaedics, Xijing Hospital, The Fourth Military Medical University, Xi'an 710032, China; ^2^Department of Orthopaedics, Fuzhou General Hospital of Nanjing Military Command, Fuzhou 350025, China; ^3^Department of Orthopaedics, Tangdu Hospital, The Fourth Military Medical University, Xi'an 710038, China

## Abstract

A microsphere composite made of poly(DL-lactic-co-glycolic acid) (PLGA), mesoporous silica nanoparticle (MSN), and nanohydroxyapatite (nHA) (PLGA-MSN/nHA) was prepared and evaluated as bone tissue engineering materials. The objective of this study was to investigate the synergistic effect of MSN/nHA on biocompatibility as well as its potential ability for bone formation. First, we found that this PLGA-MSN/nHA composite performed good characteristics on microstructure, mechanical strength, and wettability. By cell culture experiments, the adhesion and proliferation rate of the cells seeded on PLGA-MSN/nHA composite was higher than those of the controls and high levels of osteogenetic factors such as ALP and Runx-2 were detected by reverse transcriptase polymerase chain reaction. Finally, this PLGA-MSN/nHA composite was implanted into the femur bone defect in a rabbit model, and its ability to induce bone regeneration was observed by histological examinations. Twelve weeks after implantation, the bone defects had significantly more formation of mature bone and less residual materials than in the controls. These results demonstrate that this PLGA-MSN/nHA composite, introducing both MSN and nHA into PLGA microspheres, can improve the biocompatibility and osteoinductivity of composite* in vitro* and* in vivo* and had potential application in bone regeneration.

## 1. Introduction

Skeletal defects caused by severe open fractures, fracture nonunions, surgery for bone infections, and tumors remain a problem in orthopedic clinics [1, 2]. Owing to the potential for infection and bone loss, the treatment of high energy open fractures (caused by traffic accidents) [3, 4], contaminated bone fractures (Gustilo type III B and C), and long-term bone infections (chronic osteomyelitis) is a challenge, even for the best orthopedic surgeons [5, 6]. The traditional method of treating infectious bone disease is extensive debridement followed by continuous antibiotic irrigation or poly(methyl methacrylate) (PMMA) bead implantation [7]. Moreover, to repair the residual defects caused by debridement, amounts of autologous or allogeneic bone grafts are required [8, 9]. The entire treatment usually requires at least two or three operations accompanied by long-term bed rest, unbearable suffering, and considerable cost [10].

With the development of biocompatible synthetics, biodegradable polymers have been used in the fields of orthopedic and reconstructive surgery and tissue engineering [11, 12], without necessarily removing the polymeric composite after healing. Moreover, much attention has been paid to the microsphere-based composite fabricated by poly(DL-lactic-co-glycolic acid) (PLGA) [13, 14], because of its potential applications as vehicles for controlled drug delivery system and cell carrier for tissue engineering. However, the poor bioactivity and serious local inflammation are induced by the acidic degradation products of PLGA, which partially hinders its application in bone tissue engineering. An ideal strategy has typically focused on the incorporation of inorganic particles and polymer to influence the mechanical properties of composites and enhance bioavailability, biocompatibility, and osteoconduction of composites [14–16]. Similar to natural bone mineral, nanohydroxyapatite (nHA) has been studied extensively as a bioactive mineral phase additive to natural and synthetic bone biomaterials to enhance osteoblast differentiation and to improve mechanical and degradation properties of composites [15, 17]. Moreover, mesoporous silica nanoparticle (MSN) possesses high specific surface area, large pore volume, tunable pore size, and possibility of surface modification, making it superiority as carrier for loading drugs and molecules [18]. The composite microspheres of PLGA and different matrix particles (MSN or nHA) have attracted significant attention in the field of bone regeneration [19–21]. The related researches focused on two aspects: (i) the influence of nHA on biocompatibility, mechanical strength, and degradation properties of composites as well as osteoinductivity of composites and (ii) the influence of MSN on drug delivery system to increase loading efficiency, optimize drug dosage, and control drug release. Moreover, it has been reported that PLGA incorporated with MSN could enhance* in vitro* apatite mineralization and support cell adhesion, proliferation, and differentiation compared with PLGA without MSN [22].

To improve biocompatibility and expand application scope, PLGA microsphere incorporated with MSN and nHA was expected to obtain the optimum biomaterials for bone tissue engineering. In previous studies, the combination of hexagonal mesoporous silica (HMS) and hydroxyapatite (HA) in PLGA-based microspheres was developed and exhibited excellent controlled drug release properties* in vitro* [19]. However, the biocompatibility of MSN and nHA in loaded PLGA microspheres composite, especially its potential ability for bone regeneration, is still unknown.

In the present study, we prepared PLGA microspheres loaded MSN and nHA (PLGA-MSN/nHA), as well as their cylindrical composite. We hypothesized that the combination of MSN and nHA would improve biocompatibility and osteogenic potential of composite* in vitro* and* in vivo*. To test this hypothesis, the physical and chemical characteristics of the microspheres and cylindrical composites were analyzed. Subsequently, rabbit marrow stromal cells (MSCs) were cultured with cylindrical composites, and their adhesion, proliferation, and differentiation were evaluated. Finally, we evaluated the osteogenic ability of cylindrical composites by implanting them into cavity defects in rabbit femur.

## 2. Materials and Methods

### 2.1. Synthesis Methods and Characterization

#### 2.1.1. Preparation of MSN

MSN was synthesized via the typical S°I° assembly method [23]. Briefly, tetraethyl orthosilicate (TEOS, Chemical Reagent Factory, Guangzhou, China) was added to a vigorously stirred solution of dodecylamine (DDA, SSS Reagent Co., Ltd., Shanghai, China) in ethanol (EtOH) and deionized water, affording a reaction mixture of the following molar composition: TEOS : DDA : EtOH : H_2_O, 1.0 : 0.27 : 9.09 : 29.6. The reaction mixture was aged at an ambient temperature for 18 h, and the template was removed using hot EtOH at 45°C and 80°C for 1 h. Then, the final product was dried in an oven at 90°C for 1 h.

#### 2.1.2. Fabrication of Various Microspheres and Their Cylindrical Composites

Three types of microspheres (PLGA-MSN, PLGA-nHA, and PLGA-MSN/nHA) were prepared using a single emulsion solvent evaporation method [24]. Briefly, 5 g of PLGA and 1 g of MSN, nHA, or a complex of MSN and nHA (1 : 1 w/w) was dissolved in 25 mL of methylene chloride, and the mixture was sonicated for 1 min. The resultant mixture was then poured into a 0.5% poly(vinyl alcohol) (PVA, Sigma-Aldrich, Singapore) aqueous solution and stirred for 8 h. The microspheres were isolated and washed five times with deionized water. Cylindrical composites were fabricated by pouring each type of microspheres into a cylindrical mold and heating at 80°C for 2 h. The nHA (20 nm of average particle size) purchased from Emperor Nano Material Co., Ltd. (Nanjing, China) was used in this study. The PLGA with a ratio of lactic to glycolic acid monomer units of 50 : 50 was purchased from Daigang Biomaterial Co., Ltd. (Jinan, China) and has an average molecular weight of 31,000 g mol^−1^ with an inherent viscosity of 0.30 dL g^−1^ in chloroform at 30°C.

#### 2.1.3. Morphological Characterization

The morphology of the MSN dispersed in ethanol was characterized by high-resolution transmission electron microscopy (HRTEM, JEM-2010, JEOL, Japan) using an accelerating voltage of 200 kV. The porous properties of MSN were determined via the measurements of N_2_ adsorption-desorption isotherms on a Micromeritics Tristar 3000 pore analyzer under continuous adsorption condition. The Brunauer-Emmett-Teller (BET) and Barrett-Joyner-Halenda (BJH) methods were used to determine the surface area, the pore size distribution, and the pore volume. Scanning electron microscopy (SEM, 30XLFEG, Philips, Netherlands) was used to observe the morphology of the MSN and nHA particles and the three types of cylindrical composites at 10 kV after the samples were sputter-coated with a layer of gold.

#### 2.1.4. Wettability Property

The wettability of composites was evaluated by static contact-angle measurements at three different locations using a surface contact machine (OCA15, Dataphysics, Germany). Briefly, 25 *μ*L of water was dropped on the surface of each type of composite at 5 *μ*L/s (*n* = 6). Meanwhile, the contact angle was captured using a surface contact machine at a resolution of 0.01° and a side-view microscope connected to a camera (Nikon, USA). The contact angle was calculated by applying a spherical approximation using Image J 1.48 software.

#### 2.1.5. Mechanical Test

The compressive strength of the cylindrical composites (diameter = 5 mm and height = 10 mm) was analyzed in a material testing machine (MTS-858, MTS System Inc., USA) by compression in the vertical direction at a deformation rate of 5 mm/min until failure at 20°C. The compressive strength was calculated by *S* = *F*_max_/*A*, where *F*_max_ is the maximum load on the load-deformation curve and *A* is the cross-sectional area of each sample (*n* = 6).

#### 2.1.6. Degradation of the Composites

Samples of the composites were weighed and then soaked in bottles filled with 10 mL of phosphate buffered saline (PBS) solution (pH 7.40). All bottles were incubated at 37°C. The weight and pH value were measured at 1, 2, 4, and 8 weeks. The weight loss was calculated using the following equation: weight loss (wt.%) = (*W*_0_ − *W*_*t*_)/*W*_0_ × 100% (where *W*_0_ and *W*_*t*_ are the initial mass and the mass after *t* day's immersion, resp., *n* = 6).

### 2.2. Cell Culture

#### 2.2.1. Cell Culture and Seeding

Rabbit marrow-derived MSCs were used for* in vitro* experiments [25]. The cells were cultured in *α*-MEM medium (Corning, USA) supplemented with 10% fetal bovine serum (FBS, Gibco, USA), 100 U/mL penicillin, and 100 U/mL streptomycin (Sigma) at 37°C in a humidified atmosphere of 5% CO_2_ and 95% air. After sterilization by exposure to Co^60^, MSN at different concentrations (25, 50, 100, 150, 200, or 400 *μ*g/mL) were used to investigate the cytocompatibility in 6-well plates (without MSN as blank controls, *n* = 6). The three types of cylindrical composites were sterilized by soaking into 75% ethanol for 24 hours and then washing in *α*-MEM medium three times. MSCs were digested with trypsin/ethylene diaminetetraacetic acid (EDTA) to produce a cell suspension, concentrated by centrifugation at 1200 rpm for 10 minutes and seeded on the composites at 4 × 10^5^ cells. The composites with seeding cells were put into 24-well plates and incubated in a standard culture condition as preciously described. MSCs were subcultured with the composites in the same medium for one week changing the medium every 48 hours.

#### 2.2.2. Cell Adhesion on the Composites

After cells were seeded on the composites for 4 h and 24 h, the adhesion status of the cells was evaluated. Briefly, twelve composites from each group were taken from the media and gently washed with PBS three times. Then, six cell-loaded composites were fixed with 3% glutaraldehyde in PBS for 24 h at 4°C. After washing with PBS, the specimens were dehydrated in a graded series of alcohol (30–100%) followed by freeze-drying and examination under SEM. Another six composites were used for the measurement of the adhesion rate. The samples were first rinsed in 0.25% trypsin for 30 seconds and were then moved to 0.05% EDTA for 1 min. Afterward, each composite was transferred into a centrifuge tube with *α*-MEM medium containing 10% fetal bovine serum. After careful pipetting, the composite was moved from the medium, and the suspension was centrifuged at 1200 rpm. Finally, the cell precipitate was collected and subjected to cell counting. The cell adhesion rate was calculated with the following equation: cell adhesion rate (%) = *N*_*t*_/*N*_0_ × 100%, where *N*_*t*_ and *N*_0_ are the number of attached cells and seeded cells, respectively.

#### 2.2.3. Cell Proliferation on the Composites

After coculture for 7, 14, and 21 days, the viability and proliferation of MSCs were determined by WST-1 Cell Proliferation and Cytotoxicity Assay Kit (Beyotime, China). At each predetermined interval, six samples from each group were washed with PBS, transferred into a new well plate containing 600 mL WST-1 solution, and incubated at 37°C for 4 hours. At the end of the incubation time, the reaction liquid was pipetted out into 96-well plates. The absorbance was determined at 450 nm using a microplate reader (RT-6000, China).

#### 2.2.4. Osteoinductive Ability Assay

The osteoinductive ability of composite was evaluated by reverse transcriptase-PCR [26]. Briefly, the total messenger ribonucleic acid (mRNA) from the cells was isolated at 3, 7, 14, and 21 days using TRIzol (Takara). Then, 2 *μ*L of mRNA was transcribed to 20 *μ*L of complementary DNA using PrimeScriptTM RT Master Mix (cat. number RR036A, Takara). Then, 12.5 *μ*L of SYBR Premix Ex Taq II2X (cat. number RR820, Takara), 1 *μ*L of PCR forward primer (Takara) and PCR reverse primer (Takara), 2 *μ*L of cDNA, and 8.5 *μ*L of sterilized distilled water were added. Real-time PCR reactions were performed according to the following cycling program: initial denaturation at 95°C for 15 min, followed by 40 cycles of 15 s of amplification consisting of denaturation at 95°C, 45 seconds of annealing at 62°C, and an extension step at 60°C for 1 min. The primers used are listed in Table 1. For the evaluation of osteogenic differentiation, an osteogenic medium (OM, consisting of DMEM supplemented with 10% FBS, 10^−8^ M DEX, 10^−3^ M b-glycerol phosphate, and 50 mg/mL L-ascorbic acid) was used as the positive control.

### 2.3. Animal Experiments

#### 2.3.1. Implantation Surgery Procedure

Fifty-four mature New Zealand rabbits (male, 12 weeks old, 3.0 ± 0.4 kg) were subjected to implantation surgery. All animals used in this research were conducted according to the polices and principles established by the Animal Welfare Act and the National Institutes of Health Guide for the Care and Use of Laboratory. Food and water were withdrawn 12 hours before induction of anesthesia. The rabbits were anaesthetized with xylazine hydrochloride injection (intramuscular injection, 1 mg/kg) and 3% pentobarbital (intramuscular injection, 30 mg/kg). Animals were placed in lateral recumbency and right posterior limb was operated. A cavity-like (volumetric) defect of 5 mm in diameter was made perpendicularly to the bone axis into the femur condyle of both sides. The drill cavities were carefully washed to eliminate bone debris and were dried before they were filled composite. Closure of the fasciae, subcutaneous tissue, and skin was performed using absorbable sutures. A splint bandage was used to protect the operated limbs for up to 2 weeks with weekly bandage changes.

#### 2.3.2. Histological Observations

Six rabbits from each group were randomly sacrificed at 4, 8, and 12 weeks after surgery. At 14 and 4 days before sacrifice at the predetermined time point (4 weeks), the animals were labeled with tetracycline (intramuscular injection, 50 mg/kg, Sigma) and calcein (intramuscular injection, 8 mg/kg, Sigma). Then, the femur condyles were collected and fixed in 80% ethanol. After two weeks, the specimens were dehydrated in a graded ethanol series (70–100%) within a span of 24 h and then they were embedded in a methylmethacrylate (MMA) solution for 3 weeks. After polymerization of the MMA at 50°C for 24 hours, pathological sections were cut using a band saw and observed using a fluorescence microscope. The speed of new bone formation was measured by monitoring the length between the two labels over time (*μ*m/d). After observing fluorochrome double labeling, the samples were stained by Van-Gieson (VG) staining and examined by light microscopy (DM6000B, Leica Microsystems). In order to carry out histomorphometry analysis, the sections with bone tissue were pseudocolored using Adobe Photoshop CS6 software (Adobe Systems Incorporated, San Jose, CA, USA) at the same threshold and analyzed using the Image-Pro Plus system (Media Cybernetics, Silver Spring, MD, USA). The new bone formation was quantified from the pixels representing bone tissue. The rate of new bone formation was determined by the percentage of the bone area over the total implant area: BA/TA = (bone  area/total  area) × 100%, where BA is the area of the new bone formation and TA is the total area of the implantation.

### 2.4. Statistical Analyses

All data were analyzed using SPSS 10.0 software. Experiments were repeated three times, and the results are expressed as means ± standard deviations. Statistical significance was calculated using one-way analysis of variance (one-way ANOVA). Comparison between the two means was performed using Student's *t*-test. The level of significance was defined at *p* < 0.05.

## 3. Results

### 3.1. Characteristics of the Particles

**Figure 1:**
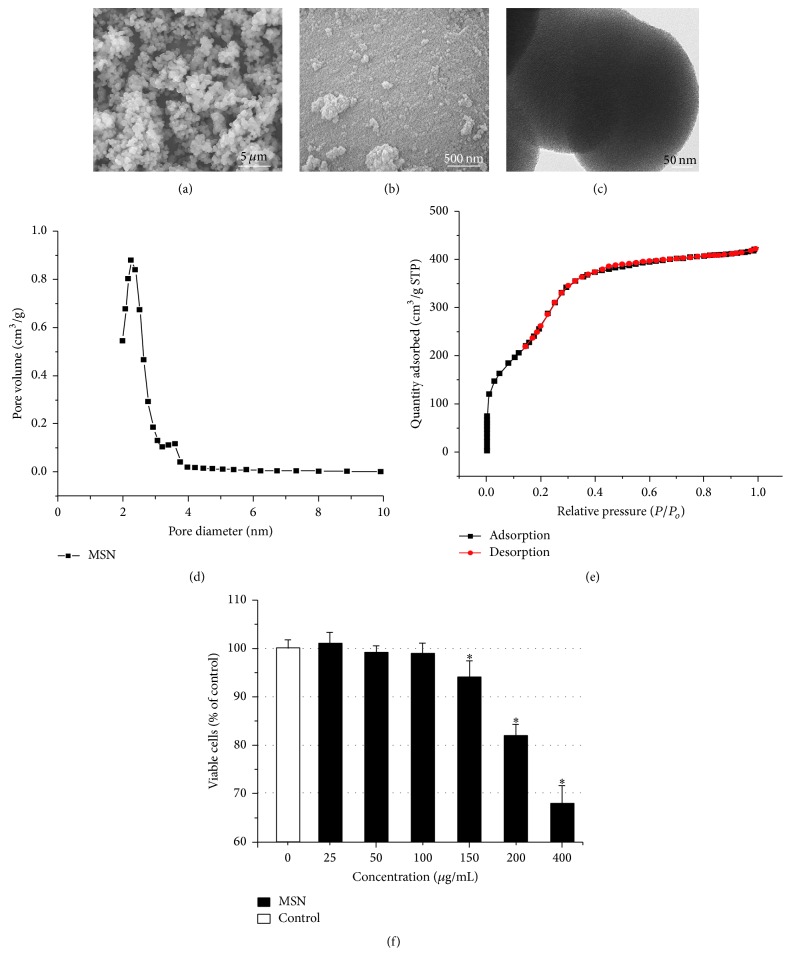
Characteristics of MSN and nHA particles. The SEM images of MSN (a) and nHA (b). The TEM image of MSN (c). The pore size distributions (d) and N_2_ adsorption-desorption isotherms (e) of MSN. The viability of MSCs incubation with MSN at different concentrations (f). ^**∗**^ *p* < 0.05 compared to the control group, and *n* = 6.

The micrographs for the MSN and nHA particles were investigated by SEM. The SEM images in Figure 1(a) demonstrated that MSN particles had an irregular sphere shape with a smooth surface, and the diameter of the particles was between 250 nm and 600 nm. The particles were naturally in contact with each other, forming a skeleton network. The size of the nHA particles was approximately 20 nm in an irregular ball shape, and some of the ball-like granules were aggregated (Figure 1(b)). HRTEM was used to study the pore structure of the MSN, and the image showed a well-defined arrangement of uniform pores (Figure 1(c)). The N_2_ sorption-desorption isotherms of MSN were shown in Figures 1(d) and 1(e). The BET surface area, pore volume, and pore size were 1142 m^2^/g, 0.65 cm^3^/g, and 2.89 nm, respectively. The capillary condensation was approximately 0.2 of *P*/*P*_0_ in the isotherms, which further confirmed the existence of a mesoporous structure and provided a pore size distribution curve calculated from the desorption branch by the BJH model. The MSCs viability assay showed that the viability of MSCs revealed a concentration-dependent decrease after incubation with MSN for 24 h. At a concentration of 150 *μ*g/mL, MSN particles significantly inhibited the growth of cells compared to blank control (Figure 1(f)).

### 3.2. Characteristics of the Composites

**Figure 2:**
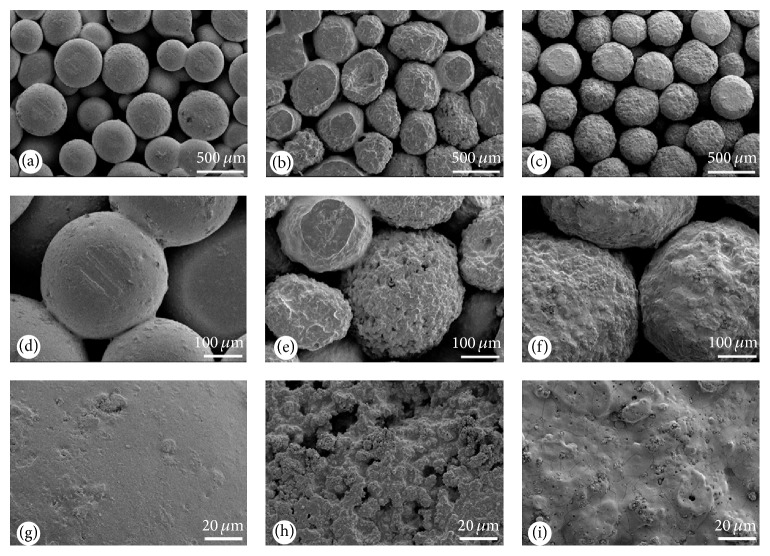
SEM images of three composites: (a, d, and g) PLGA-nHA, (b, e, and h) PLGA-MSN, and (c, f, and i) PLGA-MSN/nHA.

The structure and surface characteristics of the three composites were depicted in Figure 2. By SEM observation, the surface of PLGA-nHA microspheres was smooth and even (Figures 2(a), 2(d), and 2(g)). Owing to the close contact of the microspheres, regular tunnels were formed in the composites. In contrast, the surface of PLGA-MSN microspheres was honeycomb-like, and sharp prominences and deep holes were found in single microspheres (Figures 2(b), 2(e), and 2(h)). The roughness of PLGA-MSN/nHA microspheres was between those of the other two microspheres, and numerous micropores formed on the surface (Figure 2(c)). Based on high magnification SEM observation, the size of the micropores was approximately 1 *μ*m, and slim cracks formed between some of the micropores (Figures 2(f) and 2(i)). The average static contact angle of composites was calculated (PLGA-MSN: 92.3 ± 2.7°; PLGA-nHA: 76.0 ± 2.3°; and PLGA-MSN/nHA: 74.1 ± 1.2°). The results showed that contact angle of PLGA-MSN was significantly higher than that of the other two groups (*p* < 0.05). The compressive strength of the three composites was analyzed and the results showed that the compressive strength of PLGA-MSN/nHA (7.22 ± 0.95 Mpa) was more than twofold and sevenfold that of PLGA-nHA (3.49 ± 0.39 Mpa) and PLGA-MSN (1.08 ± 0.23 Mpa), respectively (*p* < 0.05).

**Figure 3:**
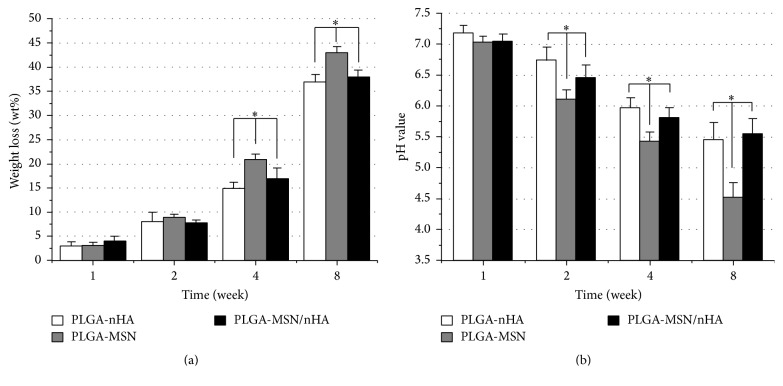
The degradation properties of three composites. (a) The weight loss (wt.%) and (b) pH values at different time point. ^**∗**^ *p* < 0.05 compared to the PLGA-MSN group, and *n* = 6.

The degradation properties of three composites were presented in Figure 3. In the early stages (1 and 2 weeks), a low weight loss (wt.%) was found for all three composites. In two weeks, no more than 10% of composite was degraded. There were no significant differences in the degradation rate in the three groups during the first two weeks (*p* > 0.05). From the fourth week on, a higher degradation rate was found in the PLGA-MSN group, and more than 20% and 40% of the composite were lost at the fourth and eighth week, respectively. The degradation rate of PLGA-MSN was significantly higher than that of the other two groups at these two time points (*p* < 0.05). There were no significant differences in the degradation rates between the PLGA-nHA and PLGA-MSN/nHA groups during the observation period. Among the three groups, the pH value of PLGA-MSN group was much lower than that of the other two groups from 2 to 8 weeks (*p* < 0.05), while there was no significant difference between the other two groups (*p* > 0.05).

### 3.3. Cell Culture with the Composites

**Figure 4:**
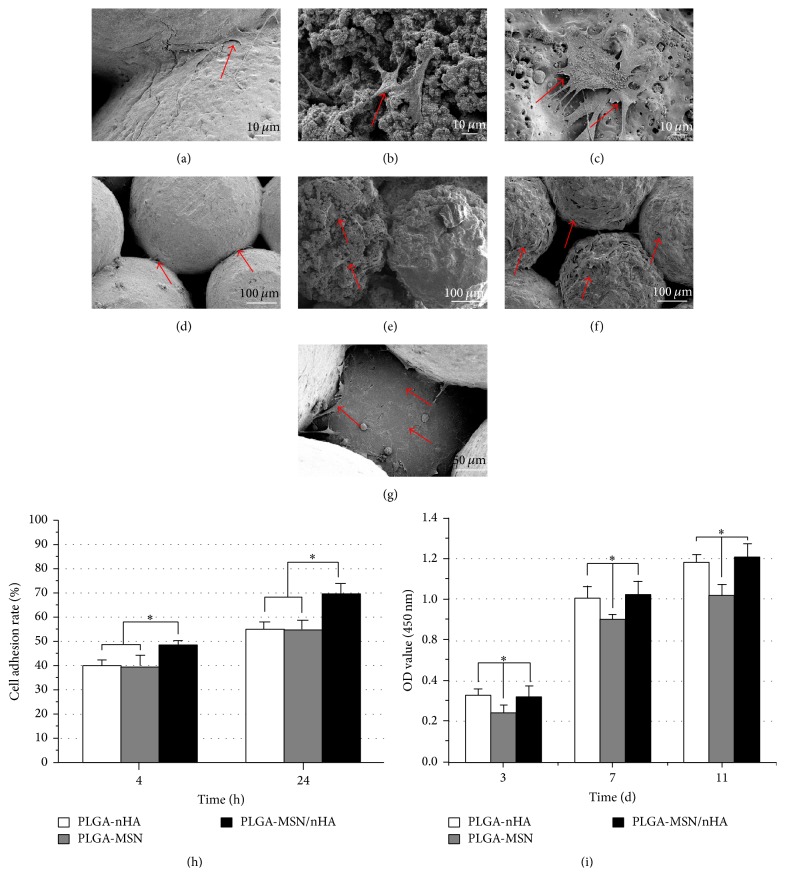
The adhesion and proliferation of MSCs on three composites. SEM images (4 hours after seeding): (a) PLGA-nHA, (b) PLGA-MSN, and (c) PLGA-MSN/nHA; SEM images (24 hours after seeding): (d) PLGA-nHA, (e) PLGA-MSN, and (f and g) PLGA-MSN/nHA; (h) the cell adhesion rate measurement; (i) the viability and proliferation of cells on the composites were measured by WST-1. The MSCs were marked with red arrows. ^*∗*^ *p* < 0.05 compared to the other groups, and *n* = 6.

The cell adhesion observed by SEM was presented in Figures 4(a)–4(g). Four hours after seeding, a few cells were found in the connected site between the microspheres in the PLGA-nHA group (Figure 4(a), red arrow). A few shriveled cells were found on the rough surface of the PLGA-MSN microspheres (Figure 4(b)). By comparison, cells with healthy shapes were spread on the PLGA-MSN/nHA composite (Figure 4(c)). Twenty-four hours later, a few cells were found on the PLGA-nHA composites (Figure 4(d)). At the same time point, there were fewer cells on the PLGA-MSN composite (Figure 4(e)). In contrast to the above two groups, numerous cells covered the PLGA-MSN/nHA composite (Figure 4(f)). Several cells were found growing into the pores of the PLGA-MN/nHA microspheres (Figure 4(g)). The results were further confirmed by the cell adhesion rate measurement (Figure 4(h)). The WST-1 detection revealed the viability and proliferation of cells on the composites from 7 to 21 days. The OD values for the PLGA-MSN/nHA and PLGA-nHA groups were significantly greater than that for the PLGA-MSN group at each time point (Figure 4(i), *p* < 0.05).

**Figure 5:**
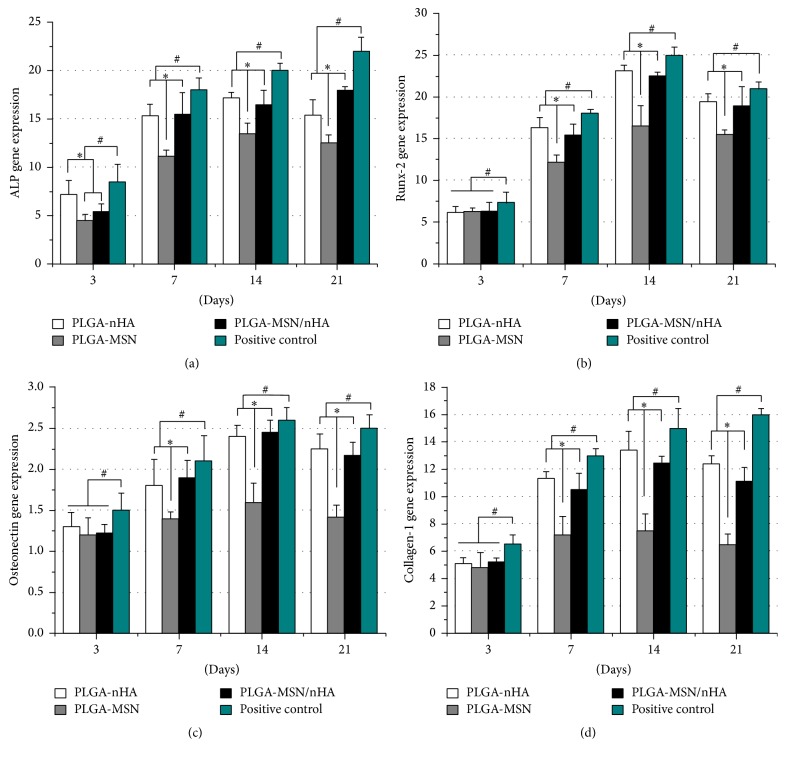
The levels of expression of osteoblast-specific genes at different time points. (a) ALP gene expression, (b) Runx-2 gene expression, (c) osteonectin gene expression, and (d) Collagen-1 gene expression. ^**∗**^ *p* < 0.05 compared to the PLGA-MSN group, ^#^ *p* < 0.05 compared to the positive control group, and *n* = 6.

The levels of expression of osteoblast-specific genes in the different groups were presented in Figure 5. In both PLGA-nHA and PLGA-MSN groups, the expression of ALP increased with time during the first two weeks and began to decrease after 14 days. In contrast, the ALP level in the PLGA-MSN/nHA group increased from 3 to 21 days. The PLGA-nHA group led to the expression of more ALP than the other two groups on the third day, while similar levels of ALP were present at the time point of 14 days between PLGA-MSN/nHA and PLGA-nHA groups. At that time, the ALP level elicited by PLGA-MSN group was lower than that for the other groups. Similar trends for Runx-2, Col-1, and osteonectin were found in the three groups: the expression of the three factors increased with time during the first two weeks and began to decrease after day 14. After the first week, the levels of the three factors in the PLGA-nHA and PLGA-MSN/nHA groups were much higher than those in the PLGA-MSN group.

### 3.4. *In Vivo* Evaluation of the Composites

**Figure 6:**
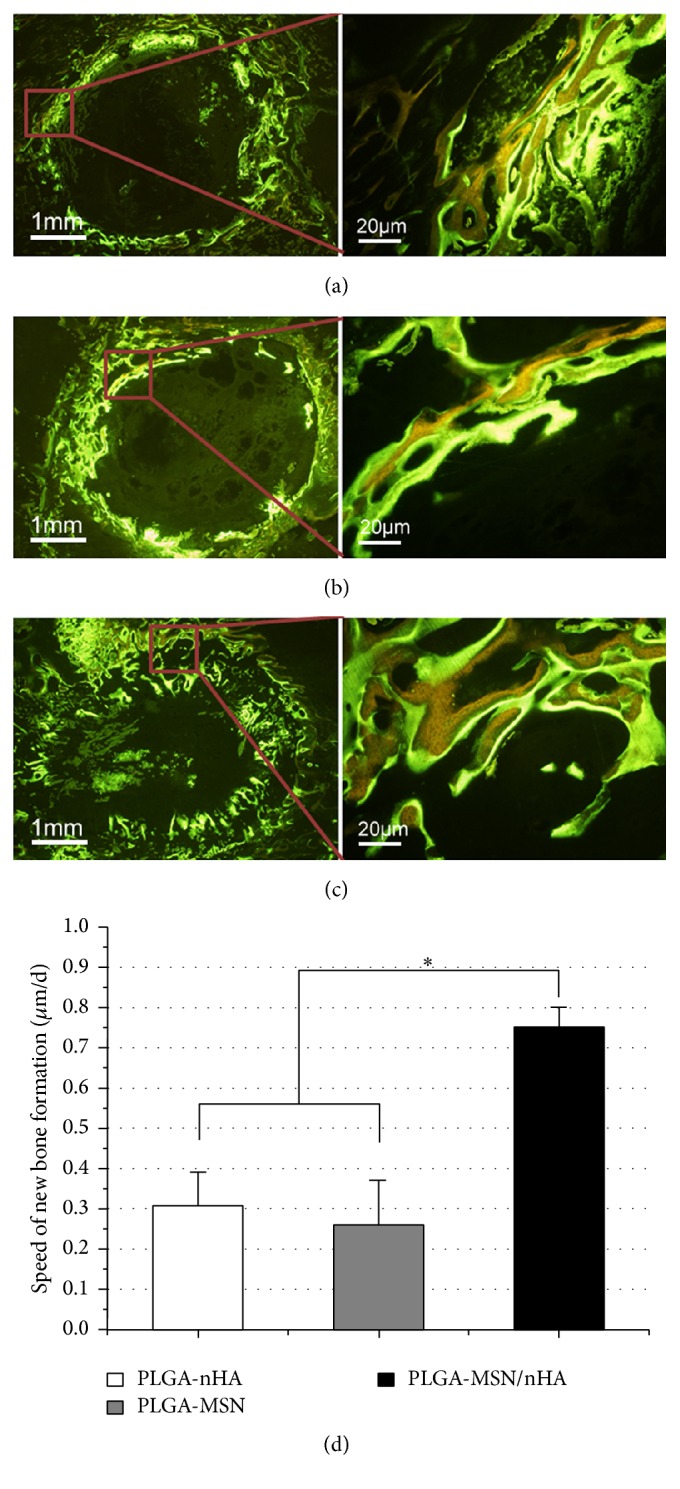
The double-labeling images and measurement after implantation (4 weeks) by fluorescence microscope. (a) PLGA-nHA, (b) PLGA-MSN, (c) PLGA-MSN/nHA, and (d) the speed of new bone formation. ^*∗*^ *p* < 0.05 compared to the control group, and *n* = 6.

The speed of new bone was detected by using a fluorescence microscope. The strong labeling of tetracycline (yellow bands) and calcein (green bands) was observed in the composite-implanted area for all three composites (Figures 6(a)–6(c)). In four weeks, newly formed bone was found in the peripheral portion of the three groups. Compared with the other two groups, the new bone of PLGA-MSN/nHA group obviously began ingrowth to the center of bone defect. The results confirmed that faster new bones have been regenerated in the PLGA-MSN/nHA group, and the speed of new bone formation in the PLGA-MSN/nHA group was more than twofold that of the other two groups (Figure 6(d), *p* < 0.05).

**Figure 7:**
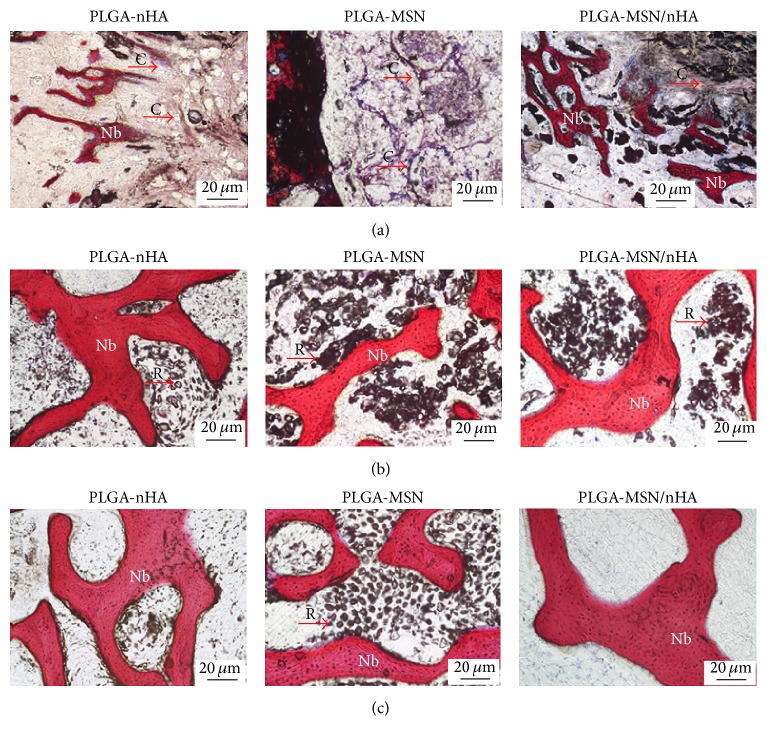
The histological images after implantation by VG staining. (a) 4 weeks; (b) 8 weeks; (c) 12 weeks. The bone, cartilage tissue, and residual materials were stained red, purple, and black, respectively (Nb: new bone; C: cartilage tissue; R: residual materials).

**Figure 8:**
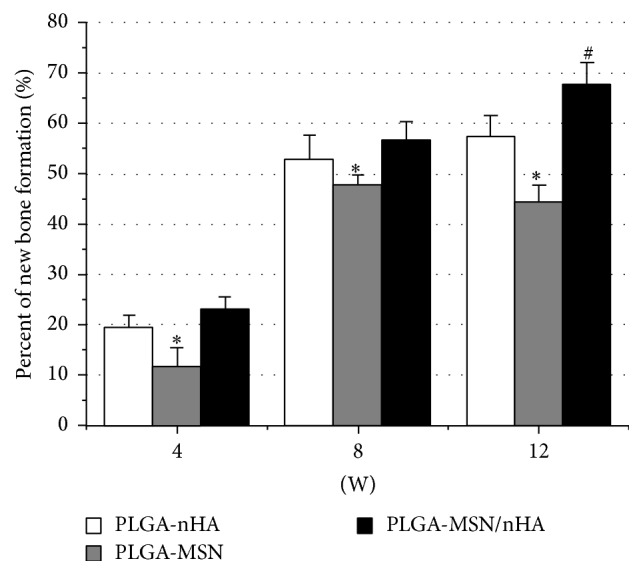
The percentage of new bone formation was measured by the histomorphometric analysis. ^*∗*^ *p* < 0.05 compared to the other two groups, ^#^ *p* < 0.05 compared to the PLGA-nHA group, and *n* = 6.

The bone, cartilage tissue, and residual materials were stained red, purple, and black, respectively, by VG staining. Four weeks after implantation (Figure 7(a)), cartilage-like tissues were found and the new bone had woven around the peripheral area of the PLGA-nHA and PLGA-MSN/nHA composites. By contrast, only cartilage tissues enveloped the PLGA-MSN composite. Eight weeks after implantation (Figure 7(b)), the formation of new bone had increased significantly in the PLGA-nHA and PLGA-MSN/nHA groups. Sponge-like bone tissues were found in both the peripheral and central zones of the bone defects. Owing to the ingrowth of new bone, the three composites began to degrade and become fractured. After twelve weeks (Figure 7(c)), woven bone combined with lamellar bone was found around the residual PLGA-nHA composite, whereas lamellar bone had formed in most of the PLGA-MSN/nHA implanted area. The defects were completely repaired with little residual materials observed. In contrast, although significant degradation of the implanted PLGA-MSN composite had occurred, a small quantity of lamellar bone was present in the area close to the host bone, and this lamellar bone was thin and irregular. Furthermore, the percentage of new bone formation (BA/TA) in the bone defect was calculated as shown in Figure 8. The BA/TA of PLGA-nHA and PLGA-MSN/nHA increased with time, whereas the BA/TA of PLGA-MSN reached its peak value and began to decrease at 8 weeks. From 4 to 12 weeks, the BA/TA of PLGA/MSN was significantly lower than that of the other two groups (*p* < 0.05). Although the BA/TA of PLGA-MSN/nHA was constantly higher than that of PLGA-nHA at the determined time points, only the difference at 12 weeks was significant (*p* < 0.05).

## 4. Discussion

Mesoporous silica has been widely investigated as a suitable carrier for a broad range of medical and biological applications, such as drug delivery, gene transfection, and cell tracing. Although numerous researches have been performed to study the* in vitro* drug loading, encapsulation efficiency, and release behavior of this mesoporous material, its* in vivo* biocompatibility has rarely been studied in bone tissue engineering.

In the present study, both the* in vitro* and* in vivo* biocompatibilities of mesoporous silica and derived microspheres composites were systematically evaluated. Using cell counting and SEM morphological examination, we found that PLGA-MSN has a small negative effect on the adhesion of MSCs (Figure 4). To determine the cause of this, the cytotoxicity of MSCs cocultured with MSN particles was evaluated. We found that a certain concentration of MSN may limit the proliferation of MSCs. The viability of cells was also decreased by high concentration of MSN, which may affect the activation of the MAPK signaling pathways [27]. The adhesion of MSCs on the PLGA-nHA and PLGA-MSN microsphere-based composites was less than that on the PLGA-MSN/nHA for various reasons. First, the biological behavior of cells can be affected by the topological structure of a scaffold surface [28, 29]. Based on the SEM observations, we determined that the surface of PLGA-MSN microspheres was honeycomb-like, and both sharp prominences and deep holes were present. The prominences were almost the same size as the MSCs, which may cause difficulty for the movement and extension of cells on the microspheres. Furthermore, the sharp rims of the prominences may damage the membrane of the seeding cells (Figure 2). However, a surface which is too smooth also has negative effects on the adhesive behavior of cells. In the SEM observations, few cells were found only in the connecting site between PLGA-nHA microspheres. Based on a report by other researchers [30], it is difficult for cells to secrete adhesion molecules when the cells are attached to the smooth surfaces of composite. Lacking adhesion molecules, the seeding cells cannot extend and are easily removed by fluid [31]. In contrast, the mild roughness of the PLGA-MSN/nHA surface was suitable for the seeding and extending of MSCs, and cells were easily loaded and grew on this composite. Second, the wettability of composite also plays an important role in the adhesive behavior of MSCs. The contact angles of water reflected the wettability of composite. The smaller the contact angle is, the higher the wettability is [32]. In this study, the contact angle of water with PLGA-MSN/nHA was significantly lower than those for PLGA-MSN, implying that PLGA-MSN/nHA had a better wettability. The surface tension between MSN and water might explain this phenomenon. In contrast, the introduction of nHA into the mesoporous silica may change the surface tension of this composite, making MSN/nHA more hydrophilic [33]. The higher hydrophilicity may also enhance the adhesion of seeding cells [34, 35].

The proliferation of cells on the different composites displayed diverse trends, which may be explained by the topological structure, degradation, and pH value of the composites. The greater roughness of the PLGA-MSN surface inhibited the seeding of MSCs. However, with continuous degradation of the composite, the surface became more even and more favorable for cell adhesion as a result of the surface being flushed and rinsed with the culture medium, causing an increase in proliferation during the first two weeks. Similarly, the smooth surface of PLGA-nHA was changed by continuous degradation in the culture medium, which promoted vigorous proliferation of cells. Although degradation also changes the surface of PLGA-MSN/nHA, cell proliferation on this composite changed less over time compared to the other groups, owing to the MSCs highly adhering to this composite during the early period (the first 24 hours). However, with increasing degradation, the pH value of the culture medium played an important role in the changes in cell proliferation on the different composites (Figure 3(b)). PLGA is a common matrix for the preparation of biodegradable implantation materials and medical devices [36, 37]. Owing to the feasibility of its use for fabrication, it can be applied as a composite to prepare diverse types of biomaterials. In previous studies, PLGA was used as the main supporting component for developing uniform microspheres to control the release of bioactive molecules and drugs [38, 39]. However, the biggest disadvantage of this material is its degradation-derived residues, which have lower pH values* in vitro* and easily evoke inflammatory reactions in the local implantation area* in vivo*. A lower pH value is thought to harm the proliferation of the surrounding cells, and the inflammatory reaction can cause the release of cytokines by the host, damaging the viable cells [37, 40]. In the present study, the cell proliferation on PLGA-MSN was significantly lower than other two groups, and acid-mediated degradation of PLGA may be the reason for this phenomenon, as evidenced by the low pH values and the higher weight loss during this period (Figure 3). To overcome the acidic degradation of PLGA, we modified the PLGA microsphere composites using nHA, which was applied in our previous studies [41]. Based on the* in vitro* and* in vivo* results of the present study, we further confirmed that the introduction of nHA could induce a microenvironment, particularly alkalization of the medium, that has a positive influence on cell metabolism, especially with respect to PLGA composites and acidic degradation products.

In addition to the neutralization of PLGA, HA also played a key role in the osteogenic activity of the material. In the present study, based on PCR reactions, we observed that the PLGA-MSN/nHA composite had better osteogenic activity than the PLGA-MSN composite (Figure 5). The nHA can promote the proliferation and metabolism of osteoblasts on the nanoscale because it has a similar structure to natural bone. Many* in vitro* studies had demonstrated that nHA could form a layer of bone-like apatite on the surface of nHA-derived materials after incubation in a simulated body fluid. Furthermore, nHA-containing particles had shown improved bioactivity and promoted better osteointegration* in vivo* studies [42–44]. However, crystalline HA degrades over a long period* in vivo,* and the presence of a large amount of undegraded HA may thus hinder or slow complete bone reconstruction [45]. In the present study, the combination of MSN and nHA was used to significantly reduce the total amount of nHA required without significantly compromising the basic biological capacity of nHA. Furthermore, this combination can improve the surface area of the derived composite for drug loading owing to the presence of larger pores and to the well-defined structure of MSN. Moreover, this combination also had good bioactivity and biocompatibility due to the nHA. Compared with nanoparticles, the special mesoporous structure, which supplies space to host a large number of drug molecules, makes MSN/nHA suitable for drug delivery.

Although the* in vitro* osteoinductive ability of PLGA-MSN/nHA is not as good as that of OM (osteogenic medium), this* in vivo* study revealed that PLGA-MSN/nHA possessed an excellent osteogenic capacity. By measuring the gap between the bands labeled with tetracycline and calcein, we found rapid bone growth in the region of PLGA-MSN/nHA, which was twofold faster than that in the control groups. PLGA-MSN/nHA was also degraded to a greater extent than the other groups, as determined by VG staining. The degradation of composites was different* in vitro* and* in vivo*. This might have some reasons. Firstly, the degradation rate of porous composites* in vivo* was faster than that* in vitro*, and acidic degradation products facilitated diffusion* in vivo*. Thus, the role of PH value was weaker* in vivo* compared with that* in vitro*. Secondly, with the bone tissue infiltration into the composites, the better osteoconductivity and mineralization of PLGA-MSN/nHA might promote the degradation and substitution. The degradation rate of composites must match the native tissue repair progress to achieve maximum effect. Prematurely degradation of composites will lead to poor induction period, while too-slow degradation will hinder new tissue ingrowth, thereby impairing tissue repair [41, 46]. Finally, a balance of new bone formation and material degradation occurred at the defective area in the presence of PLGA-MSN/nHA, and this balance has been thought to be an ideal phenomenon for bone regeneration [47]. As combined with fluorescence labeling and VG staining, we further confirmed that the newly formed tissue in the PLGA-MSN/nHA group was similar to natural bone, not only in quantity but also in quality.

## 5. Conclusion

The introduction of both MSN and nHA into PLGA microspheres can improve the biocompatibility and osteoinductivity of microspheres-based composites* in vitro* and* in vivo*. Compared to a single matrix particle (MSN or nHA), the combination of two could better support the cell attachment, proliferation, and osteogenic differentiation of MSCs and promote the regeneration of bone defects. This synergistic effect of MSN and nHA might result from their modifying of the surface morphology of microspheres, improving the mechanical strength, and adjusting the degradation.

## Supplementary Material

The distribute of these three component in composites has been determined by an energy dispersive spectrometer (EDS) equipped in SEM. In order to observe the interior of microspheres, the cross section of microspheres was performed by SEM. The Maps software was used to analyze the element distribution in the cross section. The elements distribution of the carbon (C), nitrogen (N), silicium (Si), phosphorus (P), calcium (Ca) on the surface of cross section were mapped, as shown in Figure-supplement. These three components were dispersed on the surface, and some nHA particles aggregating the spherical masses were reflected by the maps of Ca and P. The slow degradation for the introduction of nHA may result from the loading nHA could neutralize the change of the PH value of degradation solution. The acidic degradation products of PLGA could accelerate PLGA degradation.

## Figures and Tables

**Table 1:** Primers used for PCR validation.

Genes	Primer sequences and positions (5′-3′)
GAPDH-F	TGCTGGTGCTGAGTATGTGGT
GAPDH-R	AGTCTTCTGGGTGGCAGTGAT
ALP-F	CTCCATTGTCCACAGGAAATGC
ALP-R	TGTGACTGGTGACAGCAGTCTT
COL1-F	CAGCCGCTTCACCTACAGC
COL1-R	TTTTGTATTCAATCACTGTCTTGCC
RUNX2-F	CCTTCCACTCTCAGTAAGAAGA
RUNX2-R	TAAGTAAAGGTGGCTGGATAGA
Osteonectin-F	CACCATGAGAATCGCCGT
Osteonectin-R	CGTGACTTTGGGTTTCTACGC
