# Supplementary material for: Synergistic Effect of Mesoporous Silica and Hydroxyapatite in Loaded Poly(DL-lactic-co-glycolic acid) Microspheres on the Regeneration of Bone Defects
Source: Biomed Res Int. 2016 Aug 29;2016:9824827. doi: 10.1155/2016/9824827 (PMC5019907; doi:10.1155/2016/9824827)
Supplement: Supplementary file 1 — The distribute of these three component in composites has been determined by an energy dispersive spectrometer (EDS) equipped in SEM. In order to observe the interior of microspheres, the cross section of microspheres was performed by SEM. The Maps software was used to analyze the element distribution in the cross section. The elements distribution of the carbon (C), nitrogen (N), silicium (Si), phosphorus (P), calcium (Ca) on the surface of cross section were mapped, as shown in Figure-supplement. These three components were dispersed on the surface, and some nHA particles aggregating the spherical masses were reflected by the maps of Ca and P. The slow degradation for the introduction of nHA may result from the loading nHA could neutralize the change of the PH value of degradation solution. The acidic degradation products of PLGA could accelerate PLGA degradation. [file 9824827.f1.pdf]

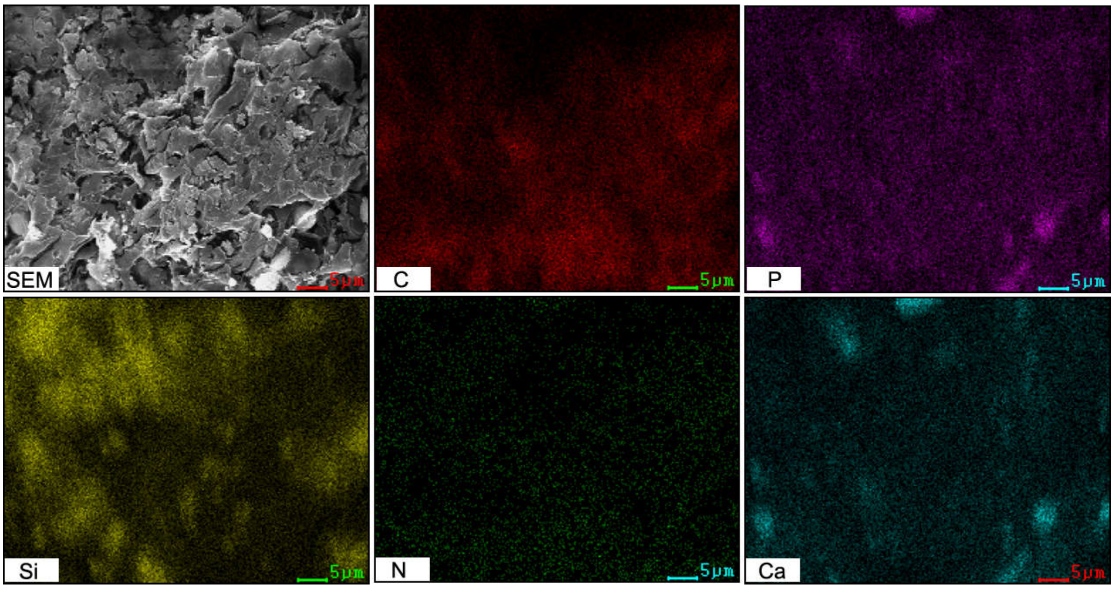

Figure-supplement The SEM and mappings of elements distribution of the cross section of PLGA-MSN/nHA microsphere. The elements distribution of the carbon (C), nitrogen (N), silicium (Si), phosphorus (P), calcium (Ca) on the surface of cross section were mapped respectively.

Notes: The maps of C and N refered to the distribute of PLGA; The maps of Ca and P refered to the distribute of nHA; The map of Si refered to the distribute of MSN.

Abbreviations: SEM, scanning electron microscope; PLGA, poly-(LD-lactide-co-glycolide) acid; MSN, mesoporous silica nanoparticle; nHA, nano-hydroxyapatite.
